# Neurodevelopmental Genes Have Not Read The DSM Criteria: Or, Have They?

**DOI:** 10.3389/fpsyt.2012.00075

**Published:** 2012-08-23

**Authors:** Valsamma Eapen

**Affiliations:** ^1^Academic Unit of Child Psychiatry South West Sydney, School of Psychiatry, University of New South WalesSydney, NSW, Australia

Most would recognize the situation: a young person presents with autistic features. While the patient himself has clear cut symptoms of autism classifiable according to any known classificatory system that is currently in use, his older brother has similar, yet different set of social deficits in that he has no friends and is being bullied due to his odd ways of communication and behaviors. Teacher reports clearly relay symptoms of Attention Deficit Hyperactivity Disorder (ADHD) in your patient and parents report that the young person fidgets and twitches. While exploring the “tic” history, your registrar reminds you that he has only had motor tics but you notice him sniffing repeatedly. When asked about the sniffing, the parents confirm that he has had it for quite some time but attributes it to allergy as it comes and goes, although with no other associated features of allergy. You also find out that a paternal uncle is “socially awkward,” dad had tics during childhood and that there is history of obsessive compulsive behaviors (OCB) in paternal aunt and grandmother. As a doctor you are concerned about the nature, degree, and functional impact of the varying symptoms on your patient's daily life in order to develop an appropriate management plan. However you are also aware of the need to communicate the nature of your patient's symptoms to other professionals who are involved in his care, to his school for attracting additional support, and to the parents who are trying to make sense of the different sets of difficulties their two boys are presenting with, and seeking answers as to why this has happened and what can be done. In the meantime your registrar is getting exhausted trying to match the criteria as per the current version of the Diagnostic and Statistical Manual (DSM) for Autism, Tourette Syndrome, ADHD, and so on, and asks whether he would qualify for a diagnosis of Tourette Syndrome now that he has been noted to have “sniffing,” a vocal tic, which together with the multiple motor tics would get the tick of approval for such a diagnosis.

## Nosology, Clinical Criteria, and Discriminant Validity

Debate on the definition, nosology, and clinical features has been re-ignited in the case of autism with the proposed revision of the criteria in DSM V (Skuse, 2012; Swedo et al., 2012). While the “nosology counts” for the prevalence rate (Fisch, 2012), clinical criteria is important for diagnosing and subtyping disorders (Witwer and Lecavalier, 2008), and diagnosis is critical for management and service provision (Nassar et al., 2009), are we loosing the essence of neurodevelopmental disorders by arguing about who should be counted in or out? In this regard, would we be justified in suggesting that the older sibling in the above case scenario is not having a clinical problem, and therefore not include him as a “case” in your genetic analysis of this family? What are the implications of the tics in this case; is it an unrelated co-morbidity, or part of the phenotypic presentation of underlying neurodevelopmental gene(s) abnormalities shared by other members of the family such as the paternal uncle, dad, paternal aunt, and grandmother? Is the ADHD in your patient linked to Autism, Tourette Syndrome, or both, or neither, and is the OCB in the female members of the family an alternate phenotypic expression of the putative Tourette gene(s), and what is the relationship of that, if any, to your patient's autistic symptoms?

Complexities in genotype–phenotype relationship and differences of opinion as to where you draw the line for a deviation or deficit to be counted as a disease condition are not new or uncommon in medicine. The diagnostic criteria for hypertension based on blood pressure level, is a case in point. Another example is that of diabetes, a clinically and genetically heterogeneous condition. While most professionals and lay public are now familiar with the two subtypes namely Type 1 and Type 2 diabetes, would it be important to recognize yet another condition called maturity onset diabetes of the young (MODY)? The answer is yes, as identifying MODY can guide management and more specifically, further subtyping of MODY based on genotypes can lead to individualized treatment such as the use of sulfonylureas in HNF1A and HNF4A mutations (Gardner and Tai, 2012). It will also have implications for identification and prognostication in family members. Clearly the solution to such heterogeneity is not changing the definition of diabetes to include or exclude the different subtypes, but a prudent and common sense approach to the “use” of the diagnosis. While communicating to the lay public, diabetes might be the best starting point, while communicating to professional colleagues, Type 1 or 2 or MODY would be appropriate and while selecting patients for pharmacotherapy research into the effectiveness of sulfonylureas, further subtyping to MODY1, 2, 3, etc. based on genotype would be warranted.

## Genetic Heterogeneity Leads to Clinical Heterogeneity

Does the case of MODY suggest a parallel to the realization that “autism” may be “autisms” and “Tourettes” may involve a spectrum of tics and related behaviors? However, the situation is more complex and challenging when it comes to neurodevelopmental disorders as they have poor discriminant validity with differing clinical phenotypes sharing the same neuro-cognitive profile or genetic underpinnings. By the same token, the same genetic abnormality can lead to no clinical symptoms, or subtle subclinical features that can only be identified through in-depth clinical or cognitive testing, or full blown clinical syndrome. Evidence is mounting on the overlap in genetic abnormalities found in different neurodevelopmental disorders (Eapen, 2011a) but how can disorders with differing clinical phenotypes share the same genetic underpinning and vice versa?

Available research data in Autism, Tourette Syndrome, and ADHD suggests the role of specific, yet overlapping neuronal circuitry. These interconnected neural systems can be linked to functionally relevant anatomic areas and pathways that influence specific cognitive or behavioral domain, the programming of which are genetically modulated during development and mediated through a range of molecular pathways and interacting neurotransmitter systems (Eapen, 2011b). Elucidating the molecular mechanisms involved in these disorders can provide an invaluable window for understanding the neural wiring that regulates higher brain functions. In the competitive and dynamic molecular environment associated with synaptogenesis it is not surprising to find that the timing and dose effects associated with disruptions, duplications, and dysregulation of the relevant genes/ligands can render varying pathogenic consequences for brain, mind, and behavior (Bottos et al., 2011). An imbalance in the excitatory/inhibitory ratio in local and extended neuronal circuits caused by genetic alterations is one example. Such events maybe sufficient but not essential, or essential but not sufficient, in producing certain behavioral profile or clinical condition, thus accounting for the variability in clinical presentations. Phenotypic variability could also be due to the genes converging on a common neurodevelopmental pathway resulting in abnormal development across disorders and broad domains and yet carrying distinct neuro-cognitive and behavioral profiles. The penetrance of the different co-morbidities may also be related to the dose effects of gene abnormality or the timing of events when different neuronal regions and circuitry are being formed, as may be the influence of gender. The “extreme male brain” hypothesis in autism (Baron-Cohen, 2002) is noteworthy here as is the suggestion that in TS, there may be a gender dependent difference in the expression of the underlying genotype with female members of the family presenting with OCB and male members with tics (Eapen et al., 1993). It has also been shown that there are sex-specific differences in the topographic segregation and functionality of brain regions and that the presence of circulating testosterone is essential for the development of the substantia nigra region in the neonatal period and to a lesser extent in the final maturation in the peripubertal period (Veliskova and Moshe, 2001). Similarly, the sex-specific imprinting of *NRXN4/CNTNAP2*, *CTNNA3*, and *LRRTM1* is known to have dramatic and variable effects on levels of gene expression and the parent-of-origin phenotypic inheritance patterns (Oudejans et al., 2004; Francks et al., 2007).

Phenotypic variability can also be affected by non-genetic factors, or “second hits” such as prematurity, perinatal trauma, injury, hypoxia, oxidative stress, infections, inflammations and autoimmunity, neural and psychosocial stressors, or other environmental modulators through epigeentics (Herbert, 2010). For example, an early environmental insult could alter the epigenetic programming with consequent changes in neural function (Zhang and Meaney, 2010). Exploring these neurobiological underpinnings can lead to a better understanding of the pathogenetic mechanisms. Again taking the example of MODY, there may be a lesson here for genotype–phenotype interactions and appropriate individualized care.

## Diagnostic Utility: Help or Hindrance?

Would not it be futile to expect the varying gene abnormalities and the manifestations of overlapping genetically determined synaptic processes that influence development to yield categorical clinical conditions? This debate is at the heart of the controversy about defining autism – one that favors a broad concept of neuro-diversity where autistic features are considered as a natural human variation and the other a narrow one. But for diagnostic purposes, there is a need to define a cut off to indicate normal vs. abnormal. By and large in psychiatry a clinical diagnosis of a disorder implies that there is significant deviation, distress, and/or dysfunction (the three D's) caused by the clinical symptoms in question. However, the prevalence of these traits or clinical disorder would depend on the definition of what is defined as a case. For example, personality traits such as being meticulous and neat and tidy are common in the general population as opposed to obsessive compulsive disorder that is clinically significant. There may be an inherent tension between determining how common it is for epidemiological purposes and for identifying cases early in the community vs. clinical diagnosis that would guide treatment. Such differences in case definition based on its utility are not uncommon, just as every case of breast cancer is not the same for the purpose of clinical diagnosis, treatment options, course, and prognosis. Also the definition of a “case” is quite different for early identification in the community such as the presence of a breast lump, vs. indication for specific chemotherapy or surgical options based on pathology report and staging. Yet for genetic research into breast cancer, neither of the above considerations would be sufficient. The same would be relevant in the case of autism, where for early identification in the community, a “breast lump” approach would be needed with anyone showing early signs and symptoms getting the benefit of further assessment and appropriate intervention, while for providing educational support, the cognitive, and functional level would need to be taken into consideration. However there is a significant dilemma when funding and support are determined by the diagnostic label rather than the adaptive functioning or other considerations.

There is no doubt that changing the criteria on blood pressure level would change the prevalence rate of hypertension in the community. However identifying those with borderline levels of blood pressure reading would be important for the purpose of prevention and early intervention. Thus it would seem that instead of changing the definition and criteria to achieve the various goals and functions, we need to strive for further refinement in categorizing and subtyping autism within the dimensional framework. It would be a great help if nosology of developmental disorders can facilitate the diagnostician through subtyping based on family history, neuro-cognitive characteristics, or symptom dimensions which would then allow early identification using behavioral telltale signs and genetic testing, choice of therapeutic options, clinical prognostication, etc., However research into autism phenotypes is in its infancy and currently there is no robust replicated evidence on reliable subtypes which made the DSM V to amalgamate the various subtypes that existed in DSM IV into a single category of ASD. Perhaps there is no evidence because we got the subgrouping wrong in DSM IV? It is hoped that progress in identifying homogenous sub types using the broad autism phenotype and linking it with genotypes and endophenotypes would offer such possibilities in the future. In the meantime DSM V has suggested the use of “specifiers,” such as severity and whether the child has intellectual disability or a language delay in the diagnostic evaluation.

Development is a dynamic process and genetically mediated deficits and consequent functional impairments involve activity-dependent synapse development that depends on postnatal learning, environment, and experiences. Thus the developmental trajectory may change as it is influenced by other factors in the early formative years of a child's life, and so will the diagnosis. This would mean that one child may lose a diagnostic label while another may pick up clinical symptoms and co-morbidities. On a positive note, this gives the opportunity for prevention and early intervention that would have the potential to alter the course of development toward a neurotypical profile and/or prevent the progression of the clinical condition by exploiting the neuronal maturation and brain plasticity. Hence, as the genetically programmed but environmentally modulated developmental process progresses, diagnosis will need to be seen as a means to assist rather than a hindrance.

## References

[B1] Baron-Cohen S. (2002). The extreme male brain theory of autism. Trends Cogn. Sci. (Regul. Ed.) 6, 248–254 10.1016/S1364-6613(02)01904-6 12039606

[B2] Bottos A. Rissone A. Bussolino F. Arese M. (2011). Neurexins and neuroligins: synapses look out of the nervous system. Cell. Mol. Life Sci. 68, 2655–2666 10.1007/s00018-011-0664-z 21394644 PMC11115133

[B3] Eapen V. (2011a). Genetic basis of autism: is there a way forward?. Curr Opin. Psychiatry 24, 226–236 10.1097/YCO.0b013e328345927e 21460645

[B4] Eapen V. (ed.). (2011b). Autism – A Neurodevelopmental Journey from Genes to Behaviour. Rijeka: InTech

[B5] Eapen V. Pauls D. L. Robertson M. M. (1993). Evidence for autosomal dominant transmission in Tourette's syndrome. United Kingdom cohort study. Br. J. Psychiatry 162, 593–596 10.1192/bjp.162.5.593 8149109

[B6] Fisch G. S. (2012). Nosology and epidemiology in autism: classification counts. Am. J. Med. Genet. C Semin. Med. Genet. 15, 91–103 22499526 10.1002/ajmg.c.31325

[B7] Francks C. Maegawa S. Lauren J. Abrahams B. S. Velayos-Baeza A. Medland S. E. Colella S. Groszer M. McAuley E. Z. Caffrey T. M. Timmusk T. Pruunsild P. Koppel I. Lind P. A. Matsumoto-Itaba N. Nicod J. Xiong L. Joober R. Enard W. Krinsky B. Nanba E. Richardson A. J. Riley B. P. Martin N. G. Strittmatter S. M. Möller H. J. Rujescu D. St Clair D. Muglia P. Roos J. L. Fisher S. E. Wade-Martins R. Rouleau G. A. Stein J. F. Karayiorgou M. Geschwind D. H. Ragoussis J. Kendler K. S. Airaksinen M. S. Oshimura M. DeLisi L. E. Monaco A. P. (2007). LRRTM1 on chromosome 2p12 is a maternally suppressed gene that is associated paternally with handedness and schizophrenia. Mol. Psychiatry 12, 1129–1139 10.1038/sj.mp.4002116 17667961 PMC2990633

[B8] Gardner D. Tai S. E. (2012). Clinical features and treatment of maturity onset diabetes of the young (MODY). Diabetes Metab. Syndr. Obes. 5, 101–108 22654519 10.2147/DMSO.S23353 PMC3363133

[B9] Herbert M. R. (2010). Contributions of the environment and environmentally vulnerable physiology to autism spectrum disorders. Curr. Opin. Neurol. 23, 103–110 10.1097/WCO.0b013e328336a01f 20087183

[B10] Nassar N. Dixon G. Bourke J. Bower C. Glasson E. de Klerk N. Leonard H. (2009). Autism spectrum disorders in young children: effect of changes in diagnostic practices. Int. J. Epidemiol. 38, 1245–1254 10.1093/ije/dyp260 19737795

[B11] Oudejans C. B. Mulders J. Lachmeijer A. M. van Dijk M. Konst A. A. Westerman B. A. van Wijk I. J. Leegwater P. A. Kato H. D. Matsuda T. Wake N. Dekker G. A. Pals G. ten Kate L. P. Blankenstein M. A. (2004). The parent-of-origin effect of 10q22 in pre-eclamptic females coincides with two regions clustered for genes with down-regulated expression in androgenetic placentas. Mol. Hum. Reprod. 10, 589–598 10.1093/molehr/gah080 15208369

[B12] Skuse D. H. (2012). DSM-5’s conceptualization of autistic disorders. J. Am. Acad. Child Adolesc. Psychiatry. 51, 344–346 10.1016/j.jaac.2012.02.009 22449638

[B13] Swedo S. E. Baird G. Cook E. H. Jr. Happe F. G. Harris J. C. Kaufmann W. E. King B. H. Lord C. E. Piven J. Rogers S. J. Spence S. J. Wetherby A. Wright H. H. (2012). Commentary from the DSM-5 workgroup on neurodevelopmental disorders. J. Am. Acad. Child Adolesc. Psychiatry 51, 347–349 10.1016/j.jaac.2012.02.013 22449639

[B14] Veliskova J. Moshe S. L. (2001). Sexual dimorphism and developmental regulation of substantia nigra function. Ann. Neurol. 50, 596–601 10.1002/ana.1248 11706965

[B15] Witwer A. N. Lecavalier L. (2008). Examining the validity of autism spectrum disorder subtypes. J. Autism Dev. Disord. 38, 1611–1624 10.1007/s10803-008-0541-2 18327636

[B16] Zhang T. Y. Meaney M. J. (2010). Epigenetics and the environmental regulation of the genome and its function. Annu. Rev. Psychol. 61, 439–466 10.1146/annurev.psych.60.110707.163625 19958180

